# Accurate peak list extraction from proteomic mass spectra for identification and profiling studies

**DOI:** 10.1186/1471-2105-11-518

**Published:** 2010-10-16

**Authors:** Nicola Barbarini, Paolo Magni

**Affiliations:** 1 Dipartimento di Informatica e Sistemistica, Università degli Studi di Pavia, Pavia, Italy

## Abstract

**Background:**

Mass spectrometry is an essential technique in proteomics both to identify the proteins of a biological sample and to compare proteomic profiles of different samples. In both cases, the main phase of the data analysis is the procedure to extract the significant features from a mass spectrum. Its final output is the so-called peak list which contains the mass, the charge and the intensity of every detected biomolecule. The main steps of the peak list extraction procedure are usually preprocessing, peak detection, peak selection, charge determination and monoisotoping operation.

**Results:**

This paper describes an original algorithm for peak list extraction from low and high resolution mass spectra. It has been developed principally to improve the precision of peak extraction in comparison to other reference algorithms. It contains many innovative features among which a sophisticated method for managing the overlapping isotopic distributions.

**Conclusions:**

The performances of the basic version of the algorithm and of its optional functionalities have been evaluated in this paper on both SELDI-TOF, MALDI-TOF and ESI-FTICR ECD mass spectra. Executable files of MassSpec, a MATLAB implementation of the peak list extraction procedure for Windows and Linux systems, can be downloaded free of charge for nonprofit institutions from the following web site: http://aimed11.unipv.it/MassSpec

## Background

Mass spectrometry (MS) has been one of the most used tools to analyze large biological molecules since the introduction of the soft ionization methods, such as electrospray ionization (ESI) and matrix-assisted laser desorption/ionization (MALDI). For this reason MS is increasingly being used in proteomics. In particular, MS is exploited in proteomics to cope with two main tasks: determination of proteomic profiles of several samples for differential studies (profiling - clinical proteomics) and the identification of proteins or characterization of post-translational modifications (PTMs) in a biological sample (identification - biological proteomics) [1].

The widely used protocols for protein identification or characterization of PTMs are PMF (Peptide Mass Fingerprinting) and PFF (Peptide Fragment Fingerprinting) [2-4]. MALDI-TOF is the platform generally exploited for PMF, whereas different platforms are used for PFF (e.g., ESI-Q/TOF, MALDI-TOF/TOF or ESI-FTICR) [5-7]. Electron capture dissociation (ECD) combined with ESI-FTICR is the most powerful technique for characterization of intact proteins [8-10]. For all these platforms the determination of the monoisotopic masses of the detected ions (i.e., monoisotoping and charge state determination) is one of the main steps of the data analysis and interpretation; in fact, every meaningful conclusion of the proteomic studies depends on the extracted peak list.

On the other hand MS has been used also for protein profiling. The aim of these studies is to discover the differences between the proteomic profiling of samples representing different conditions (e.g., healthy/pathological). In this field, apart from the new approaches of quantitative proteomics (ICAT, SILAC, etc.), an important MS platform is SELDI-TOF that differs from MALDI-TOF for the presence in the sample preparation of one or more protein chips (e.g., IMAC, WCX, etc.), expressly developed to selectively capture proteins [11]. Spectra obtained from different subjects are generally considered in these studies. It is well recognized that the main problem in differential analysis is the high number of features (m/z) in each spectrum, known as high-dimensionality-small-sample problem [12]. Several approaches have been proposed to extract a reasonable number of significant features from the whole spectrum, on which, subsequently, different classification methods can be successfully applied. Peak list extraction is one of these approaches.

All the types of spectra described above, although quite different, require a similar analysis pipeline starting from the raw spectrum to the peak list. This procedure for peak list extraction is usually composed by the following steps: preprocessing, peak detection, peak selection, monoisotoping and charge state determination (or deconvolution). The last operation of monoisotoping and charge state determination is the crucial phase of the procedure. During this step the monoisotopic masses of the detected biomolecules are determined by the analysis of the isotopic distributions (IDs) present in the mass spectrum. In addition to the procedures embedded in the software distributed with the instruments (MassLynx-Waters, DataExplorer-AppliedBiosystems, etc.), several algorithms have been developed for peak list extraction. They are generally based on the comparison between each real ID (RID) in the spectrum and the corresponding theoretical ID (TID). Apart from few approaches which try to find a global optimal solution (called non-greedy) [13,14], the major part of the algorithms for feature extraction are iterative subtractive fitting methods [15-18]. Then, the TID at certain mass is built generally considering an artificial sequence with the same mass of the unknown sequence but composed by only an "average" amino acid, called Averagine proposed for the first time by [19]. Such TID can be computed by one of the algorithms proposed in the literature [20-22].

In the case of the SELDI-TOF spectra, both the low resolution power (RP) of the spectrometer and the high values of the analyzed masses can determine an overlapping between the isotopic peaks of an ID. Unfortunately, it can happen in these conditions that the only detectable isotopic peak is the most likely one (i.e., the highest peak of an ID which corresponds to the most abundant configuration of the molecule in nature); so it is necessary to use suitable models to determine the monoisotopic mass of the molecules. The major part of features reduction/identification procedures applied on such SELDI-TOF spectra do not exploit some chemical model, but they are based on algorithms like local maxima finding or wavelet transform [23-28].

In this paper, a novel algorithm for peak list extraction from both low and high resolution mass spectra is presented. It includes and integrates in the same algorithm several ideas already reported in the literature by different authors and never used together. The most interesting innovative features adopted in the proposed procedure can be summarized in the following three points. First, it is suitable to analyze with good performance several kinds of mass spectra from both identification and profiling studies. In fact, the double model, on which the peak extraction algorithm is based, permits to analyze spectra characterized by a wide range of RP. RP of a mass spectrometer typically ranges from 500 for the low resolution MALDI/SELDI-TOF instruments to 15.000 for TOF analyzers, but can reach even 100.000 for FT-ICR and 200.000 for Orbitrap [29]. Note that, nowadays the only model-based approach applicable to both low and high resolution spectra is that proposed in [30]. Other original features of the presented approach concern the strategy for building the TIDs. The procedure includes a TID prediction model more accurate than those used in the previous published algorithms [15,17], exploiting the factional Averagine approach proposed in [14]. At the same time, it permits the use of reference amino acids on which to build the TID alternative to classical Averagine [19], including an updated version of the Averagine itself designed within this project on a recent release of the SwissProt database. This helps to handle the wide variability of the amino-acid sequences of the detected proteins/peptides. This feature is particularly useful to manage IDs with shape significantly different from the classical Averagine-based one. No previous work raised the problem of updating the Senko's Averagine with the new available proteomic information. Finally, an important original contribute of this paper is the successful attempt to manage the overlapping between IDs and, at the same time, to correct the misalignment among scans/replicates. The management of the overlapping IDs is the most difficult and frequent problem that the peak list extraction algorithms have to cope with. The proposed approach exploits the coefficient of correlation through the so-called subspectra (in the following named replicates) as suggested by [31]. In addition, the algorithm proposes, for the first time, to exploit in the peak detection the coefficient of correlation among peaks calculated on the subspectra. Subspectra/replicates are a great available source of information often unused by the most widely used tools. For example, in order to produce a single SELDI/MALDI-TOF spectrum, many scans (i.e. laser shots) are performed in different positions of the sample spot, everyone generating a scan spectrum which can be considered as a replicate. All these scan spectra are usually simply summarized by the Vendors' tools to obtain the representative mass spectrum.

The performances of the different versions of the algorithm were tested on a reference low resolution SELDI-TOF dataset (CAMDA2006), on a high resolution SELDI-TOF dataset from NCI (National Cancer Institute), on some MALDI-TOF mass spectra of the Aurum database and on a reference ESI-FTICR ECD spectrum. Finally, because the optional functionalities of the here proposed algorithm are fully effective only when many replicates/subspectra are available, an additional original MALDI-TOF spectrum with many stored scans was considered.

## Methods

In this section the model-based procedure for peak list extraction from different kinds of mass spectra is presented.

The input data of the algorithm can be a single proteomic mass spectrum (i.e., a vector of m/z values together with their intensities). For a successful application of the procedure the input signal must be acquired in "profile mode" (i.e., the quantity of substance analyzed has to be enough to generate the RIDs in the spectrum). Moreover, the algorithm can analyze also a set of subspectra composed by different scans of the same sample or by correlated spectra of different samples (e.g., in profiling studies). These multiple spectra will be indicated as replicates. When M replicates are available, first a vector of the N m/z values common to all the spectra is derived and then an NxM matrix, which contains the intensities for all the spectra, is computed. The sum of the intensities on the same row of this matrix is considered as the representative spectrum of the experiment. Obviously, when only a single spectrum is available, the representative spectrum coincides with the acquired one.

The procedure presented in this paper requires a preliminary elaboration [32], described in the first subsection, consisting in: i) the definition of the three reference amino acids (*Amms*) to exploit for TID calculation, i.e. the Averagine proposed in [19], the Leucine, and an updated version of the Averagine based on the Release 55.2 (08-Apr-2008) of the SwissProt database; ii) the computation and storage of the IDs for a series of artificial sequences composed by such reference amino acids in order to speed up the computation of the TIDs. These two operations do not belong to the analysis of mass spectrum, but the produced output will be necessary for each analysis.

In addition to the choice of which reference amino acid to use for the analysis, the algorithm requires to set the following two parameters which depend on the input data: (i) an initial guess of the RP (*RPin*) of the instrument around which the effective RP (*RPest*) will be estimated by the algorithm itself; the maximum number of charges (*ZMax*) which reasonably can ionize every molecule according to the used platform.

Typically *ZMax *should be set to 1 for MALDI spectra, 2 or 3 for SELDI spectra (for which some molecules bi- or tri-charged could be present), whereas for an ESI-FTICR ECD spectrum (like the one presented in this paper) an higher value is suitable (e.g. 30).

The procedure for peak list extraction, discussed in the remaining part of this section, includes the following steps: i) preprocessing; ii) computation of all the possible TIDs for each m/z in the representative spectrum exploiting the results of the preliminary elaboration; iii) estimation of the effective RP of the instrument; iv) peak detection on the representative spectrum; v) calculation of an intensity threshold which is dependent on the signal and is used to remove noise peaks; vi) monoisotoping and charge state determination during which, for each ID recognized in the spectrum, its monoisotopic mass, its intensities and its ionizing charge are extracted and reported.

Moreover, two further functionalities to manage the overlapping among IDs and to correct the misalignment of the replicates were developed and will be presented.

### Preliminary elaboration: creation of the reference isotopic distributions

The procedures for peak list extraction are usually based on the comparison between the real spectrum and TIDs. The method for TIDs calculation is based on the table of the isotopic abundances and on reference amino acids.

#### Calculation of an isotopic distribution

The first problem is the definition of a method able to compute the ID of a given complex molecule. Recently, a method for a fast computation of IDs has been developed [22], improving the reference algorithm presented in [21]. However, because the calculation of the IDs is performed once during the preliminary elaboration, speed is not one of the crucial requirements; so, a simple iterative algorithm, based on a matrix approach introduced in [20], was adopted and implemented.

To better understand how the algorithm works, let us consider the molecule *X_m_Y_n_*, whose elements X and Y have a known isotope abundance distribution in nature (e.g. 99% of isotope 0 and 1% of the isotope 1). The basic idea is to compute the distribution of interest through an iterative procedure that builds the molecule of interest atom by atom. So, the ID of the molecule *X_m_Y_n _*is obtained after m+n-1 iteration steps. In each step, the vector A, representing the ID of the "current" molecule, is updated by considering the vector B, representing the ID of the next atom, in the following two substeps: i) the matrix C of the joint distribution of A and B is computed simply by multiplying the two distribution vectors; ii) the probability of the isotopic configurations with the same mass weights are summarized and the ID of the "new" extended molecule is stored again in A. For example,

$$\begin{array}{l} \left[\begin{array}{c} 0.99\\ 0.01\\ 0\\ 0\\ .. \end{array}\right]\left[\begin{array}{cc} 0.99 & 0.01 \end{array}\right]=\\ =\left[\begin{array}{cc} 0.9801 & 0.0099\\ 0.0099 & 0.0001\\ 0 & 0\\ 0 & 0\\ .. & .. \end{array}\right]\to \left[\begin{array}{c} 0.9801\\ 0.0198\\ 0.0001\\ 0\\ .. \end{array}\right]\\ \text{A B}=\text{C}\to \text{A} \end{array}$$

#### Computation and storage of reference IDs

The simple procedure just described cannot be straightforwardly applied because the atomic composition of each charged biomolecule detected by the mass spectrometer is not known and only the monoisotopic mass is available. The ID can be approximated by the ID of a virtual sequence having the same monoisotopic mass but composed by a suitable number of residues identical to the reference amino acid. This ID, called TID, can be computed by the matrix approach described above. In this work, the following three reference amino acids are alternatively considered.

1. The Averagine proposed in [19] by Senko et al.:

$$Ave=C_{4.938}H_{7.758}O_{1.477}N_{1.358}S_{0.042}=111.054Da$$

2. The Leucine, a real amino acid, which has the most representative atomic composition among all the other residues, because it is the most frequent amino acid in nature [33] and it shares its formula with Isoleucine:

$$Leu=C_{6}H_{11}O_{1}N_{1}S_{0}=113.084Da$$

3. An update version of Averagine, that we have built on the Release 55.2 of SwissProt:

$$AvU=C_{4.949}H_{7.833}O_{1.473}N_{1.361}S_{0.038}=111.125Da$$

The calculation of a TID, give a monoisotopic mass, is computationally very expensive, so it cannot be performed for every ID detected in a spectrum. Moreover, it could be necessary to apply the reference amino acid procedure on monoisotopic masses which are not an exact multiple of the weight of the reference amino acid. For these reasons a preliminary elaboration is performed and a great number of TIDs are stored. For every reference amino acid, all the artificial sequences of n residues, with n between 1 and a number such as the mass of the sequence is about 20000 Da (mass range in real world experiment is often under this boundary), are generated and their IDs calculated. For computational reasons, only the first 30 isotopic peaks starting from the monoisotopic one are stored. The same operation is performed also keeping 170000 Da as limit (upperbound of some SELDI spectra). Subsequently, when spectra are analyzed, the stored IDs lower than 20 kDa are considered by default by the peak list extraction procedure. However, if the range of the input mass spectrum overcomes 20 kDa then the stored IDs lower than 170 kDa are automatically exploited.

### Preprocessing of the spectra

A baseline correction algorithm is applied twice on all the input spectra to remove the systematic components of the noise [34]. Other popular preprocessing algorithms, such as smoothing, normalization or alignment, are not used in this paper in order to preserve as much as possible the information contained in the acquired spectra, even if a sort of smoothing is indirectly performed as result of the summarization of the replicates. However, additional smoothing algorithms, like Lowess or Savitzky-Golay, were implemented and could be applied too [35,36]. Conversely, normalization must not be applied, so that the contribution of each subspectrum (replicates) in the representative spectrum is proportional to its total ion current. A procedure for spectra alignment will be discussed as an optional functionality. When replicates are available, a quality control is applied by removing the spectra with a coefficient of correlation against the representative spectrum lower than 0.4.

### Computation of all the possible TIDs

For each of the m/z values in the representative spectrum a vector of predicted intensities of the first 30 isotopic peaks are computed by interpolating the results of the preliminary elaboration. This operation is repeated for each of the considered reference amino acids.

### Resolution power estimation

Given the spectrum to analyze, it is necessary to know the RP of the instrument which generated the data. The user usually knows the nominal RP of the instrument, but often it does not exactly correspond to the RP observed in the data. It happens because the RP of the instrument gets worse over time; moreover the misalignment error among the subspectra, which are summed to build the representative spectrum, contributes to the worsening of the final RP. For this reason, a procedure able to estimate the really observed RP has been designed.

The representative spectrum is divided into a predefined number of intervals (e.g., four intervals), everyone containing the same amount of points (m/z values), and for every interval the m/z with maximum intensity is considered. This maximum is considered as the most likely peak of an ID and the procedure for monoisotoping and charge state determination (subsequently described) is applied by varying the RP around the *RPin *parameter. A series of different RPs are tested on the maximum of each interval, obtaining the corresponding fitting scores. So, the mean among the fitting scores obtained for every RP is calculated and the RP to which corresponds the minimum of such means is finally selected as estimated RP (*RPest*).

### Peak detection

**Figure 1:**
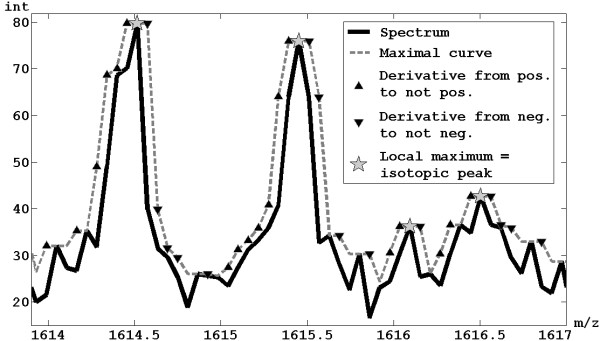
**Peak detection**. Example of application of the method for peak detection by local maxima finding.

The proposed methodology calculates the positions of all the local maxima in the representative spectrum, considering them as possible isotopic peaks. In particular, the maximum value is calculated within a moving window of width (*m/z*)*/RPest *for each m/z values of the representative spectrum; the set of these maxima forms a "maximal curve". A local maximum of the maximal curve is detected as central point of a region starting where the derivative passes from a positive value to a not positive and ending where the derivative passes from not negative to a negative value. An example of peak detection is shown in Figure 1. For simplicity, the error is calculated assuming that all the ions are mono-charged.

### Building of the intensity threshold

**Figure 2:**
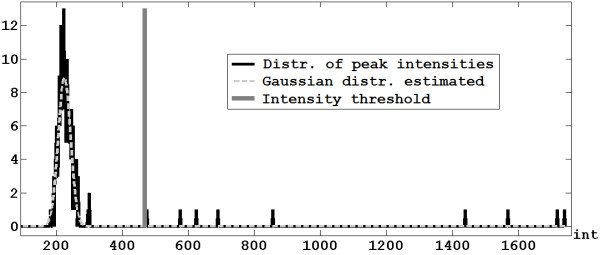
**Intensity threshold**. Computation of the intensity threshold for one of the selected windows.

A signal-dependent intensity threshold to filter out noise peaks is derived in the following way. The distribution of the intensities of the local maxima calculated in the previous step is considered and the noise peaks are assumed to be spread in gaussian way (Figure 2). The spectrum is analyzed by a moving window: the window width depends on the mass of the central m/z value, and it is inversely proportional to the density distribution of the peptide/protein masses of a reference database (e.g., Release 55.2 of SwissProt). A gaussian curve (with mean *μ *and standard deviation *σ*) is estimated for each window, and the value of the intensity threshold referred to the m/z at the center of such window is set to *μ*+2*σ*. This limit is chosen to minimize as much as possible the false positives (i.e., noise peaks that are considered as real isotopic peaks). Finally, the full intensity threshold curve is obtained by linear interpolation of the threshold values computed for all the windows. All the peaks whose intensity is lower than the threshold curve are discarded, while the remaining peaks are included in the vector *Peaks*.

### Monoisotoping and charge state determination

**Figure 3:**
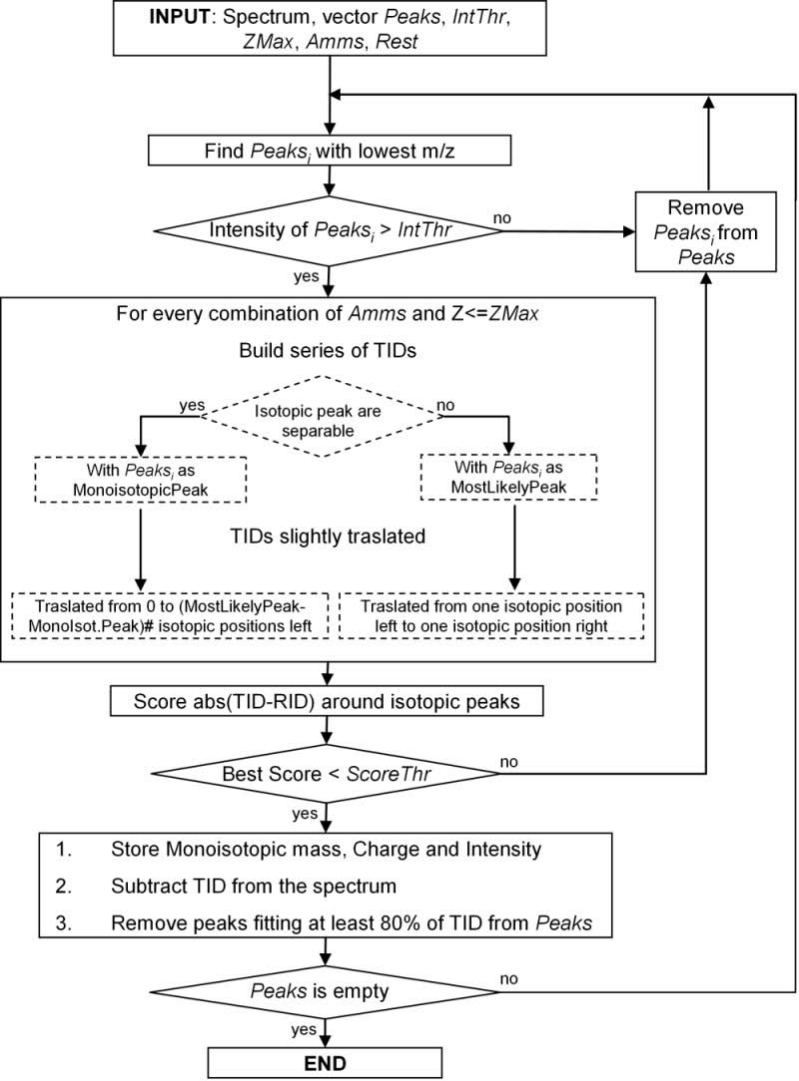
**Flowchart**. Flowchart of the algorithm for monoisotoping and charge state determination.

Starting from the vector *Peaks*, the list of the detected IDs is generated applying the following steps (Figure 3).

**Figure 4:**
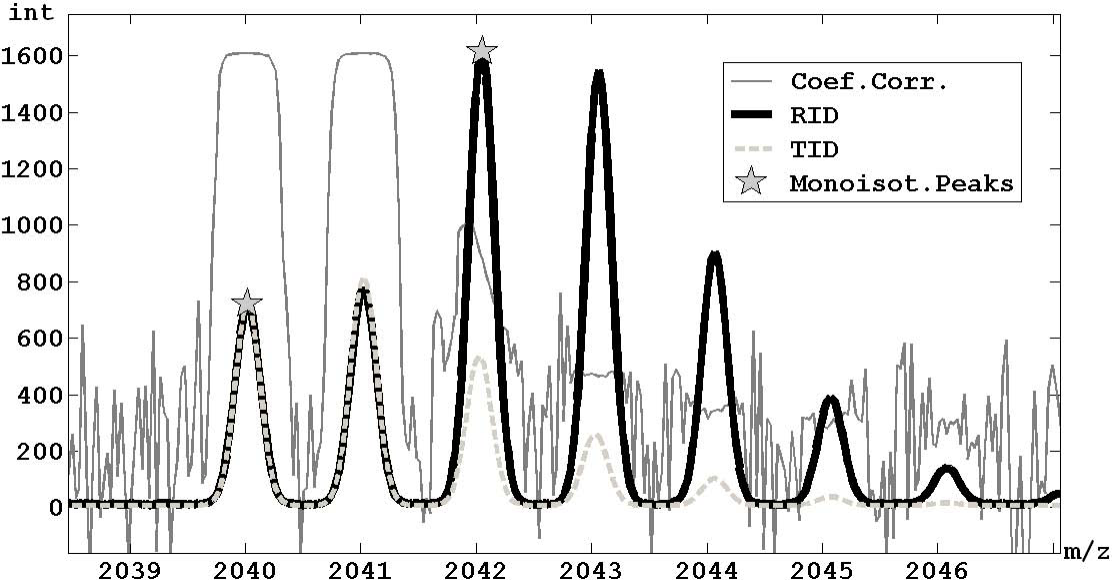
**Overlapping management**. Example of extraction of an ID with monoisotopic peak at 2040 m/z, which is partially overlapped with another ID with monoisotopic peak at 2042 m/z, exploiting the method to manage overlapping IDs.

**Figure 5:**
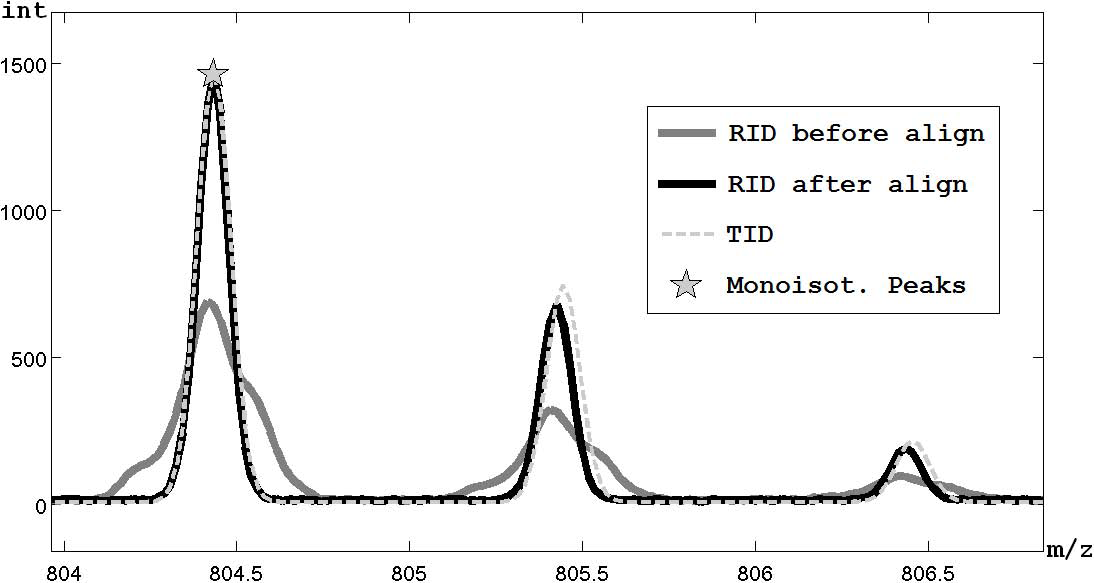
**Correction of misalignment**. Results of the correction of misalignment on a representative spectrum.

1. The peak *Peaks_i _*with the lowest m/z among all those in the vector *Peaks *is considered as reference peak.

2. If *Peaks_i _*is over the intensity threshold (see the previous subsection), the algorithm proceeds with the next step, otherwise it is removed from the vector *Peaks *and the algorithm restarts from the step 1.

3. The charge *Z_j _*of the selected molecule is set to 1 and further incremented until *ZMax*.

4. Given *RPest*, *Z_j _*and *Peaks_i_*, if *Mz_i_/RPest <*1*/Z_j_*, i.e. isotopic peaks are well-separated, the reference peak is the monoisotopic one, otherwise the most likely peak is taken as reference and the monoisotopic one is defined by a suitable regression model.

5. The first reference amino acid (*Amms_k_*) is selected among those considered.

6. Given *Z_j_*, *Amms_k _*and the monoisotopic mass associated to *Peaks_i_*, the related TID, computed as already described, is retrieved. The smallest peaks of TID are removed, keeping only those peaks whose intensity is over one thousandth of the maximum of the TID. A region of the representative spectrum around the considered *Peaks_i _*is selected by setting its two boundaries before the first isotopic peak and after the last considered one (two examples are shown in Figure 4 and 5).

7. TID is refined adding a gaussian bell with mean in correspondence of every isotopic peak and standard deviation *σ *= *Peaks_i_*/(2.35482*RPest). This formula was derived by considering that the RP is defined as m/Δm, where Δm is the minimum distance between the apexes of separable peaks (usually measured as the Full Width at Half Maximum - FWHM) and that the standard deviation *σ *is correlated to FWHM as follows: FWHM = 2$\sqrt{2ln2}$*σ *≈ 2.35482 *σ*. The absolute intensity of each peak is calculated by setting the reference peak of the TID equal to the intensity of the *Peaks_i _*in the representative spectrum and consequently scaling the whole TID, considering also as local residual baseline the lowest intensity of the region.

8. TID is shifted within the region by following two types of movements, maximizing the goodness of fitting of TID against the real spectrum. The first movement has step-width equal to 1*/Z_j _*and then, for each of the tried steps, TID is further slightly shifted for every m/z values within an interval whose range dependent on *RPest*. Note that, when the reference peak is considered by the algorithm as the monoisotopic one following the rule of the step 4, it could occur that the real monoisotopic peak is under the intensity threshold previously defined and then such a peak has been wrongly removed from the vector *Peaks*. In this case, the considered peak could be really the most likely one, so, the TID, as first movement, has to be shifted to the left for how many are the isotopic positions between the monoisotopic and the most likely peak at the mass under investigation. On the other hand, when the most likely peak is chosen as reference in step 4, the first movement of TID is performed by a shift of one isotopic peak position on the left and one position on the right. This is done because of the error which could be made by the regression model in predicting the position of the most likely peak.

9. To evaluate the goodness of fitting of the TID against real data, a score is computed as the sum of absolute differences evaluated in correspondence of some points appropriately chosen as follows. If *ZMax *= 1 only the position of the reference peak and some points at the left of the estimated monoisotopic peak are taken into account to avoid errors due to ID overlapping. Conversely, when *ZMax >*1, it is necessary to evaluate the fitting of the whole TID (i.e., the points around all the isotopic peaks) in order to correctly estimate the effective charge state of the ion. In the case of ESI, because low ionizing charges are less probable, the fitting score is increased when the number of the charges decreases.

10. If no more reference amino acid has to be considered, the algorithm proceeds with the next step, otherwise the next reference amino acid is selected and the algorithm restarts from the step 6.

11. If the tested charge *Z_j _*value is already equal to *ZMax*, the algorithm proceeds with the next step, otherwise *Z_j _*is increased of one unit and the algorithm restarts from the step 4.

12. The TID obtaining the best score (the lowest one) is selected. If this best score is over a predetermined score threshold (e.g., 0.35), *Peaks_i _*is removed from *Peaks *vector. If such score is under such a threshold, the TID is stored in the peak list and subtracted from the real spectrum. In particular for each TID the corresponding monoisotopic mass, charge and intensity are considered. The sum of the intensities of all the peaks of TID is taken as intensity, instead of only the intensity of the most likely peak. The reason is that it is considered a more accurate measure of the real quantity of molecules reaching the detector (considering together all the isotopic variants).

At last, all the peaks involved in the computation of the score and in correspondence of which the TID fits well with the real spectrum (greater than 80% of the intensity of the RID peak) are removed from the *Peaks *vector.

13. If there are still some remaining peaks in *Peaks*, the algorithm restarts from the step 1, otherwise the peak list is fully generated.

### Optional functionalities

When replicates are available, the procedure has two further functionalities that exploit the correlation among subspectra. These two functionalities are useful both during RP estimation and the final step of monoisotoping and charge state determination.

#### Management of overlapping IDs

As previously described, the fitting between a TID and the real spectrum is evaluated by the sum of the absolute differences in correspondence of the isotopic peaks, but in case of overlapping between IDs some troubles have to face (an example of overlapping IDs is shown in Figure 4). In order to successfully manage also the IDs overlapping, the score is computed by weighting the differences on the basis of the coefficient of correlation between the reference peak and each peak of the TID, calculated through the replicates (see Figure 4). In this way, an overlapping peak has a low weight because it has a low correlation with the reference peak. Moreover, during the step 12 of the algorithm described above, not only the peaks well-fitted by the TID will be removed from the initial vector *Peaks*, but also all the peaks characterized by an high coefficient of correlation with the reference peak.

#### Correction of replicates misalignment

Given the region identified in the step 6 of the procedure for monoisotoping and charge state determination, every replicate is appropriately shifted within this region in order to be well-aligned to the representative spectrum. The shift is performed in order to maximize the correlation between every replicate and the representative spectrum; in particular the correlation is calculated only in correspondence of the most correlated points. Then, the representative spectrum is recalculated in the region under investigation, considering the best alignment of replicates. The further operations will be performed on the new representative spectrum. This operation is applied on every analyzed RID. An example of misalignment correction is presented in Figure 5.

## Results and Discussion

The performance of the overall procedure was tested on spectra characterized by different values of RP from both profiling and identification studies. Before reporting and commenting results, the main comparison strategy usually adopted in the literature in the different contexts will be discussed.

### Data from identification studies

In this context all the procedures for peak list extraction are usually evaluated on spectra obtained from sample of known proteins or peptides, but so far no standard protocol has been established. The measure more commonly used is the precision (or in same case the false discovery rate - FDR) or more frequently the number of peaks correctly extracted [14-16,18,30]. Some of these algorithms are further evaluated considering the impact on the protein/peptide identification; this can be estimated by analyzing the improvement of the score used to rank the candidate peptides/proteins [18,30]. Finally, some works analyze the performance of the different methods pointing out the attention in a specific region of the spectrum particularly interesting or also evaluating the running time [14-16,30].

Therefore, the procedure here proposed was tested also on high resolution data from identification studies following these guidelines. Some MALDI-TOF spectra of the Aurum database, an ESI-FTICR ECD spectrum and an additional MALDI-TOF spectrum for which many replicates were stored were considered. Performances were measured in term of peak retrieval precision and, in the case of PMF studies, considering also the improvement in term of the Mascot protein identification score. The ESI-FTICR ECD spectrum was evaluated also showing the results in a particularly complex region of the spectrum. Some evaluation in term of running time will be reported in the conclusions.

#### MALDI-TOF spectra

The method proposed was tested on some MALDI-TOF spectra of Aurum database obtained from the analysis of 20 well-known proteins [37]. The Aurum database is a collection of both PMF and PFF mass spectra of well-known proteins (i.e. 300 Genway proteins) acquired by MALDI-TOF and MALDI-TOF/TOF platforms. It is intended to be a reference set of spectra available to train and test algorithms [38].

The proteins having at least four replicate spectra were selected to test our procedure also with optional functionalities. Then, a further selection criterion was adopted in order to consider only spectra which permitted a good protein identification in order to be able to distinguish between true positive (TP) and false positive (FP) peaks and calculate the precision. The spectra satisfying these criteria where related to 20 different proteins.

The theoretical masses of all the possible peptides generated from the digestion of each protein were derived in silico, being the identity of the 20 proteins known, as well as the enzyme used for digestion and all the possible PTMs produced by the experiment [39].

Thanks this information, the algorithm was evaluated following two different perspective.

• First, the algorithm was evaluated in terms of precision (equals to 1-FDR). In fact, peak list extraction can be considered as a classification problem, characterized by some positive classifications (the peaks extracted by the algorithm, TP+FP), among which the TPs are all the extracted peaks whose monoisotopic masses match with those of the theoretical peptides. So, the overall precision is calculated as #TP/(#TP+#FP). To make comparable the performance of different algorithms, we considered the same number of extracted peaks. In particular, because the two manufacturer-provided software (MassLynx and DataExplorer) does not automatically put a significance cutoff in the ranked peak list extracted from the spectra, we consider for all the software the a number of peak equal to that provided by MassSpec algorithm (for PDIA1 spectrum 129 with the basic version and 111 using optional functionalities, while for Aurum dataset the mean number of peaks was 142.7 and 139.9, respectively).

• Then, for data coming from PMF experiment, the accuracy of the peak list extraction procedure was evaluated in terms of improvement of the reliability of the protein identification. Mascot tool was exploited for the protein identification starting from the peak list extracted from a spectrum [40]. The protein with highest score is usually considered as the unknown protein in the sample. The absolute value of the score can be a good measure of the reliability of this identification, but a better measure is represented by the distance (difference of score - ΔSc) between the first and the second protein in the Mascot ranking. The bigger this difference the greater is the reliability of the identification, i.e. the probability that the first protein is really the unknown protein of the sample.

As reported in Table 1, the basic version of MassSpec algorithm shows a better performance than the two reference software tools (i.e., DataExplorer and MassLynx) in terms of both precision and ΔSc, while the optional functionalities do not improve significantly the results because of the low number of available replicates.

**Table 1:** Results on Aurum dataset. Precision (percentage) and absolute number of matching IDs (within brackets) and ΔSc calculated on MALDI-TOF data by MassSpec algorithm, DataExplorer (DE) and MassLynx (ML).

	Basic			Incl. optional funct.			
	**MassSpec**	**DE***^**a**^*	**ML***^**b**^*	**MassSpec**	**DE***^**a**^*	**ML***^**b**^*	

*Aurum*	20.3%(28.6)	19.6%(27.6)	17.5%(24.5)	21.3%(29.5)	19.6%(27.2)	17.7%(24.3)	Prec
	101.7	97.3	92.7	91.5	96.7	93.6	ΔSc
*PDIA1*	27.1%(35)	26.4%(34)	24%(31)	32.4%(36)	29.7%(33)	27%(30)	Prec
	161	147	130	196	161	120	ΔSc

Default parameters: *^a ^*advanced baseline correction, peak detection and peak monoisotoping; *^b ^*mass measure and TOF transform.

Mean values are reported for 20 proteins of the Aurum dataset

#### A MALDI-TOF spectrum with many scans

To fully test the real benefits of the optional functionalities on real data, a MALDI-TOF mass spectrum with 161 available scans (replicates) was analyzed. This spectrum was obtained from a spot containing trypsin-digested PDIA1_MOUSE protein. After the quality control check, 94 scans were remained, allowing an excellent working of the optional functionalities.

Results, reported in Table 1, still show better performance of MassSpec algorithm compared to the two reference tools. Moreover, there is a good improvement of the performance in terms of both precision and ΔSc using the optional functionalities compared to the basic version of the algorithm.

#### An ESI-FTICR ECD spectrum

Our algorithm was tested also on a complex high resolution ESI-FTICR spectrum previously published [9]. It is a plasma ECD spectrum of carbonic anhydrase (m/z 500-2200) recorded by Cornell 6T FTMS. The results were compared to the ones obtained by two reference algorithms for FTMS analysis: AID-MS and Hardklor [15,16]. The assignment of the fragments to the peak list was done by MS-Product [41]. The 611 IDs, extracted by AID-MS and presented in [15], were taken as reference. Therefore, the comparison of these results was performed by considering the 611 peaks with highest intensities extracted by both MassSpec algorithm and Hardklor.

Table 2 shows that the performances of MassSpec algorithm are similar to AID-MS in terms of precision, while Hardklor seems to have worse results.

**Table 2:** Results on plasma ECD spectrum of carbonic anhydrase.

	*Fragm*: *a, b, c, y, z*	*Incl*. − *H*_2_*O*, −*NH*_3_
AID-MS *^a^*	73.1%(447)	78.2%(478)
MassSpec	72.0%(440)	78.4%(479)
Hardklor*^b^*	61.2%(374)	66.4%(406)

Precision and absolute number of assigned IDs for different algorithms on a plasma ECD spectrum of carbonic anhydrase, by considering only theoretical fragments a, b, c, y, z and including water and ammonia losses

*^a ^*Published results. *^b ^*Default parameters.

**Figure 6:**
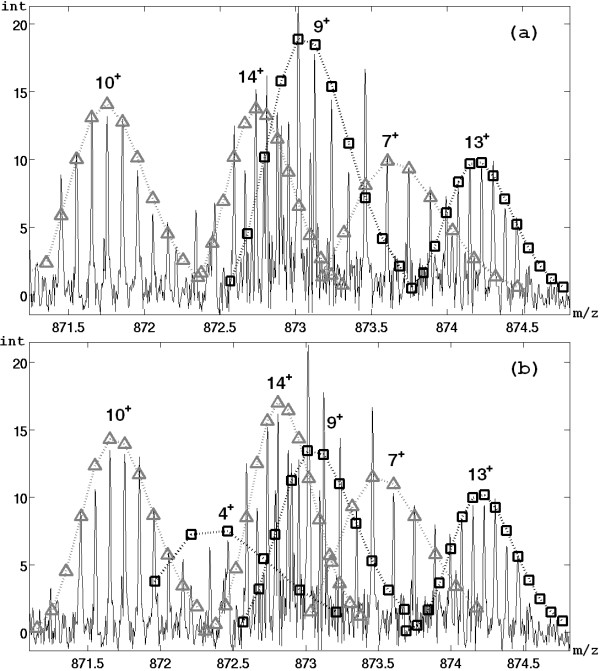
**IDs extracted from a region of the plasma ECD spectrum of carbonic anhydrase**. Comparison of performance between AID-MS and MassSpec algorithm using a region of the plasma ECD of carbonic anhydrase. Squares and triangles are neighboring IDs identified by AID-MS (a) and MassSpec algorithm (b). The monoisotopic masses of all identified IDs are shown in Table 3.

Figure 6 and Table 3 show the results of the assignment of the fragments for a region of the spectrum (already discussed in [15]). All the IDs identified by AID-MS are extracted by MassSpec algorithm, while Hardklor shows the worst performances in this region. Moreover a further ID corresponding to a fragment (monoisotopic mass 3483.632 and charge 4) is extracted only by MassSpec algorithm.

**Table 3:** Results on a region of plasma ECD spectrum of carbonic anhydrase.

AID-MS		MassSpec		Hardklor			
***charge***	***monomass***	***charge***	***monomass***	***charge***	***monomass***	***theor.monomass***	***fragment***

10+	8702.456	10+	8701.530	10+	8703.4353	8702.287	*c*_77_
		4+	3483.848			3483.632	*a*_30 _*− NH*_3_
14+	12197.186	14+	12198.298	14+	12198.153	12197.976	*C*_108_
9+	7844.078	9+	7844.121	9+	7845.099	7843.908	*c*_69_
7+	6105.177	7+	6104.238			6105.060	*b*_55 _− *H*_2_*O*
13+	11344.866	13+	11344.983			11344.611	*c*_100_

Monoisotopic mass and charges for the extracted IDs shown in Figure 5

### Data from profiling studies

In this context methods for peak identification (or more generally for feature reduction) are usually applied on datasets of SELDI-TOF spectra coming from subjects in different conditions (e.g. healthy and pathological) and evaluated indirectly on the basis of the accuracy reached by different classifiers that exploit the extracted features [24,27,42]. Moreover, in the majority of the works the algorithms are tested more directly on spectra obtained from mixture of known polypeptides [26,28,31]; in this way some metrics typically adopted to compare predictive tools like sensitivity and FDR can be calculated. The relationship between sensitivity and FDR is often used to assess the performance of a peak list extraction algorithm. Then, the approach proposed in this paper was tested on the CAMDA 2006 dataset [43] which permits to calculate sensitivity and FDR, being composed of spectra coming from a known peptide mixture. Unfortunately, this dataset is composed only by low resolution spectra and no high resolution profiling data obtained from known mixture of protein are available in literature. For these reasons the "ovarian cancer" dataset provided by NCI [44] was considered to test the procedure also on high resolution spectra and, in this case, the algorithm was evaluated by the classification accuracy.

#### Low resolution SELDI-TOF spectra

The CAMDA 2006 dataset [43] is composed by low resolution SELDI-TOF mass spectra. Data are from the All-in-one Protein Standard sample (Ciphergen Biosystems Inc.) acquired using a Ciphergen NP20 chip. Seven polypeptides are present in the sample and correspond to the following mass values: 7034, 12230, 16952, 29023, 46671, 66433 and 147300. The optional functionalities of the proposed algorithm can be used and tested because 64 replicates were stored and are available.

In order to assess the performance of the algorithm, the sensitivity and the FDR were calculated (as described in [28]) by varying some parameters of the algorithm, mainly regarding the intensity threshold. The sensitivity was defined as the number of identified true positive peaks divided by the total number of real peaks. Since the mixture contained 7 polypeptides, assuming a maximum of 3 charges for polypeptides the true peaks result to be (at least from a theoretical point of view) 21. It is important to note that the sensitivity so calculated can be underestimated because some peaks (especially those triply charged) could be not really present in the acquired spectrum.

The FDR was defined as the number of wrongly identified peaks divided by the total number of identified peaks. An extracted peak was considered as wrong if it is not within a tolerance window of *±*1% around the m/z of one of the polypeptides in the mixture.

**Figure 7:**
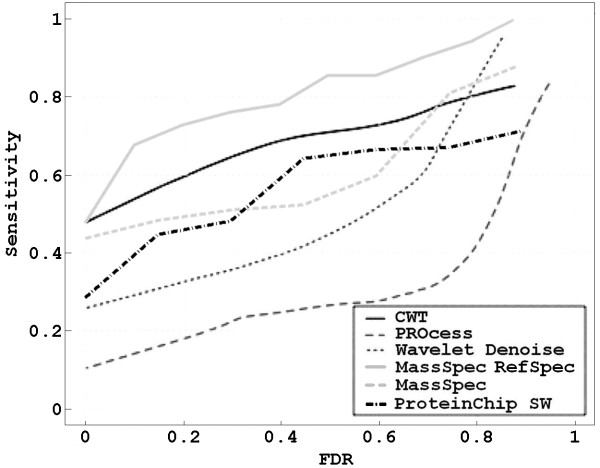
**FDR-sensitivity relationship on CAMDA dataset**. Comparison of the proposed algorithm with other four reference algorithms based on FDR-sensitivity relationship assesed on the CAMDA 2006 dataset.

Results were summarized by plotting the FDR-sensitivity curve. In particular, two curves were computed: one obtained considering independently every spectrum of the dataset (Figure 7 - dashed line), another one obtained considering all the 64 spectra in the dataset as replicates and building the reference spectrum (Figure 7 - solid line). Previous works demonstrated that the use of a reference spectrum (e.g. the mean spectrum) improves the performance of peak detection methods [26]. Moreover, in this case, it is possible to exploit also the optional functionalities of the here proposed procedure.

FDR-sensitivity curves were computed also by using Chiphergen ProteinChip software [23], that extracts the peak list following a two-step procedure: first a peak detection algorithm was applied on each spectrum, then the most frequent detected peaks are grouped by a clustering procedure. Sensitivity and FDR values were obtained by varying some parameters of the peak detection algorithm and the clustering step.

In Figure 7 these curves were compared with those computed in [28] by using three others reference algorithms: the one implemented in the Bioconductor PROcess package, the wavelet denoising method proposed by [25] and the approach based on the continuous wavelet transform (CWT) presented just in [28].

Figure 7 demonstrates that the performance of MassSpec on single spectra is comparable with the other reference algorithms specifically designed for low resolution data, but the proposed procedure clearly outperforms all the others when it is applied on the reference spectrum. It provides the highest sensitivity values for different FDR values; the greatest improvement (mainly respect to wavelet denoise, PROcess and ProteinChip software) are obtained at the lowest FDR values. That is an important result because, since in profiling studies the peak list extraction phase is useful also for feature reduction, it is important to achieve good performance by minimizing the number of false peaks to limit the high-dimensionality-small-sample problem always present [12].

#### High resolution SELDI-TOF spectra

This dataset of high resolution mass spectra is made publicly available by the Center for Cancer Research of NCI (National Cancer Institute) [44]. It consists of 216 mass spectra (121 ovarian cancer patients and 95 healthy women) obtained from serum samples. The goal of the study was to discover biomarkers for ovarian cancer in the low molecular weight serum proteome, amplified by binding to circulating carrier proteins such as albumin [11,45-47].

**Figure 8:**
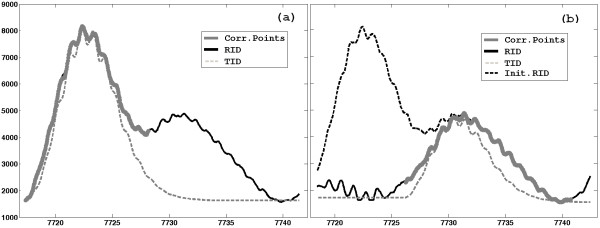
**Extraction of two overlapping IDs**. The extraction of two overlapping IDs is helped by the application of the optional functionalities that exploit the correlation among replicates.

Every spectrum is composed by 373401 different m/z values which are the candidate biomarkers (or features of the classification problem). The procedure proposed in this paper considered the different spectra in each group of subjects as replicated and it was applied on the sum spectrum by setting the maximum number of charges equals to 2 and *RPin *equals to 8000. By using only the basic version (without optional functionalities) good results in term of feature reduction were reached: 617 different IDs were extracted both singly and doubly charged. Whereas by applying the full procedure 560 IDs (singly and doubly charged) were extracted. The application of the optional functionalities, especially that for the management of the ID overlapping, is very useful, given the great complexity of the high resolution MS profile of the entire low molecular weight human serum (see Figure 8). It was feasible thanks the great number of spectra (subjects) composing the dataset.

The intensity of the most likely peak of every ID was stored for every spectrum in order to use this data for the subsequent differential analysis by classification algorithms finalized to discover a single or a pattern of biomarking biomolecules for ovarian cancer. Two different machine learning approaches (OneR and PART), implemented in the software Weka [48], were chosen and applied to 26 peaks further selected by a feature reduction procedure present in Weka ("GreedyStepwise" search with "CfsSubsetEval" evaluator [49]). Performances were evaluated within a leave-one-out cross-validation framework. The choice of the classifiers was based on their capability to build a simple diagnostic model easily to understand and interpret.

The simple OneR algorithm selects the minimum-error attribute to predict the class (i.e., using only one attribute), discretizing continuous attributes [50]. The selected feature is the 8597.5 peak and the accuracy achieved is quite good, i.e. 87.5% (see [27]).

PART is a method for generating "PARTial" decisional trees. In particular, it builds a partial C4.5 decision tree in each iteration and makes the "best" leaf into a rule [51]. The accuracy obtained by leave-one-out cross-validation is 90.3%. The list of rules built by the complete training set contains the 4 rules shows in Table 4 and uses only 4 different features.

**Table 4:** List of the rules obtained on the "ovarian cancer" dataset by PART.

IF 8597.51 ≤ 8.741252	THEN No Cancer
IF 8597.51 ≤ 14.880854	
AND 7057.04 ≤ 7.94864	
AND 6849.60 ≤ 4.599669	THEN No Cancer

IF 4895.26 ≤ 3.519758	THEN No Cancer

ELSE Cancer	

For example, the rule "IF. 8597.51 ≤ 8.741252 THEN No Cancer" means that if the intensity at m/z 8597.51 is less or equals to 8.741252, then the patient to which the spectrum is related can be classified as healthy

Better classification accuracy values were reached by other classifiers (e.g., 94.4% by RBF - radial basis function - network and 93.5% by SMO-SVM - support vector machine), but results of these methods are more difficult to interpret and to use as meaningful diagnostic models. All these results obtained starting from the peak list generated by MassSpec are not worse than those obtained in the literature applying other feature extraction algorithms on the same dataset [27,42]. The main advantage of using the peak list instead of other data mining techniques to extract relevant features is that peaks can be associated to IDs and with further efforts possible to a list of peptides/proteins. This step from a clinical point of view is not irrelevant.

## Conclusions

The best way to extract useful information from proteomic mass spectra is to generate the peak list composed by the monoisotopic mass, the charge and the intensity of each detected biomolecule. This is not a trivial task and its precision can be crucial to reach useful results both in identification and in profiling studies. Notwithstanding, peak list extraction is widely applied to identification spectra, whereas in profiling studies the step of feature extraction is often performed by other data mining procedures like wavelet transform or simple local maxima finding, by ignoring the biochemical background.

In the procedure proposed in this paper a mass spectrum is modeled as a sum of the IDs generated by the detected ions and determined by the RP of the instrument, the mass of the molecule and the acquired charge. Because the algorithm for feature extraction is based on a double model, considering as reference both the monoisotopic and the most likely peak, it has been possible to extend the peak list extraction to the analysis of profiling spectra, making the procedure independent of MS platform in terms of resolution power.

The main goal of the proposed procedure is to improve the peak list extraction operation in terms of precision rather than reducing the computational time. Due to the complexity of the mass spectrum of a biological sample, the algorithm for peak list extraction had to cope with many problems. (i) The high level of noise is faced by a sophisticated method to calculate a signal-dependent intensity threshold. (ii) The wide variability of the amino-acid sequences of the proteins/peptides detected is handled by an improved prediction model for TIDs and by the possibility to use more than only one reference amino acid to build the TIDs. (iii) Moreover an update version of classical Averagine has been designed; the obtained results demonstrates a good impact in using this residue because, among the three reference amino acids, it was often the best choice (for Aurum data, about 70% of IDs were extracted choosing updated Averagine). (iv) The problem of the IDs overlapping, which more than the others affects the performance of peak list extraction, is solved by exploiting the correlation among replicates (when available). (v) This correlation is used also to correct the misalignment among replicates.

The algorithm was tested on data from both identification and profiling studies. The peak lists extracted from spectra obtained from 20 proteins of Aurum database prove that the algorithm often produce better results than two popular software (DataExplorer and MassLynx) both in term of precision of IDs extracted and in term of ability to improve the quality of the protein identification performed with Mascot. The application of the proposed procedure on a MALDI-TOF spectrum of which many scans (replicates) were available demonstrates that the optional functionalities, when applicable, can significantly improve the performances. The algorithm was applied also on a complex ESI-FTICR ECD spectrum to test the performances for both ESI data (high number of ionizing charges) and very high RP (about 60000). The precision of MassSpec algorithm is comparable and, in some cases, better than some sophisticated algorithms previously published. If scans had been stored and made available, such good results could be certainly improved by using the optional functionalities, which could be very useful mainly to manage overlapping IDs, that are very frequent in so complex spectra.

The results obtained on a low resolution SELDI-TOF dataset (CAMDA 2006) are better than the results reached by some reference algorithms specifically designed for low resolution spectra, showing the best comparative performance at the lowest FDR values. The high values of accuracy reached on the "ovarian cancer" dataset using the attributes extracted by the proposed procedure demonstrate that it helps to extract suitable information also from high resolution profiling data.

The algorithm was evaluated also on simulated mass spectra to test its performance in terms of sensitivity, as well as precision. The obtained results were promising but are not included in this paper for the clearness of the presentation.

Although the final goal was to achieve an high accurate peak list, the running time of the basic version of MassSpec algorithm is comparable with other similar software: e.g. the mean time spent for peak list extraction on Aurum spectra was 25.7 seconds using a quite standard Sun workstation (two AMD Opteron 200 Series CPUs Model 252 - 2.6 GHz and 4 GB of RAM). Obviously run time deeply dependent on the use of optional functionalities and on the value of parameter *ZMax*. Then it decreases using only one reference amino acid (about 9 seconds on Aurum data).

Future developments of the procedure will concern its application on LC-MS data. So far, the method for peak list extraction was successfully applied on both LTQ and OrbiTrap proteomic mass spectra (respectively zoom and full MS1 scans) to improve precursor mass determination in PFF experiments. Furthermore, in this kind of experiments, we planned to implement an iterative procedure to select groups of subspectra neighboring in the chromatogram as replicates, for applying optional functionalities.

## Availability

The proposed procedure was implemented in MATLAB (7.4.0) and compiled with MATLAB Compiler toolbox (4.6). The graphical user interface was developed using the MATLAB GUIDE tool. The software tool, called MassSpec, exploits some functions included in the Bioinformatics (2.5) and Curve Fitting (1.1.7) toolboxes. It is distributed with the runtime MATLAB libraries and does not requires any MATLAB license. A beta-version of the tool is available for Windows and Linux systems together with the installation instruction at the web site http://aimed11.unipv.it/MassSpec. The tool reads two kinds of input: a single mass spectrum, acquired in profile mode, both in ASCII data file format (a list of m/z values with the corresponding intensities) and in mzXML format, or a whole dataset of replicates as a folder containing a file for every spectrum. Some test data (a dataset of 20 MALDI-TOF scan spectra) are available with the software tool to evaluate the proposed procedure. The implemented algorithm could be used to analyze mass spectra obtained by one of the considered MS-platforms (i.e. MALDI/SELDI-TOF and ESI-FTICR ECD) regardless manufacturers.

## Authors' contributions

NB led the design and the implementation of the overall procedure, its evaluation and drafted the manuscript. PM supervised the design of the procedure, its evaluation and finalized the manuscript. All authors read and approved the final version of the manuscript.
